# Experimental Investigation on Aerodynamic Performance of Inclined Hovering with Asymmetric Wing Rotation

**DOI:** 10.3390/biomimetics9040225

**Published:** 2024-04-09

**Authors:** Mengzong Zheng, Liansong Peng, Guanting Su, Tianyu Pan, Qiushi Li

**Affiliations:** 1 Research Institute of Aero-Engine, Beihang University, Beijing 100191, China; zhengmengzong@buaa.edu.cn (M.Z.); pantianyu@buaa.edu.cn (T.P.); liqs@buaa.edu.cn (Q.L.); 2 China Academy of Information and Communications Technology, Institute for the Integration of Informatization and Industrialization, Beijing 100190, China; pengliansong@caict.ac.cn; 3 School of Energy and Power Engineering, Beihang University, Beijing 100191, China; 4 Key Laboratory of Fluid and Power Machinery, Ministry of Education, Xihua University, Chengdu 610039, China; 5 Engineering Research Center of Intelligent Air-Ground Integration Vehicle and Control, Ministry of Education, Xihua University, Chengdu 610039, China

**Keywords:** model experiment method, dragonfly, inclined hovering, aerodynamic performance

## Abstract

This study presents a model experiment method that can accurately reproduce the flapping motion of insect wings and measure related unsteady aerodynamic data in real time. This method is applied to investigate the aerodynamic characteristics of inclined hovering, which distinguishes it from normal hovering by having asymmetric wing rotation during the two half strokes. In the study of the aerodynamic influence of the downstroke rotational angle, it is found that the rotational angle affects lift generation by changing the angle between the wing surface and the horizontal plane in the mid-downstroke. When the wing is almost parallel to the horizontal plane in the mid-downstroke, the vortex structure can maintain structural integrity and a large magnitude, which is conducive to the generation of high lift. In the study of the aerodynamic effect of the upstroke rotational angle, the windward conversion mechanism is proposed to explain the influence of the upstroke rotational angle on the direction and magnitude of thrust. Obtaining the rotational angle that is most conducive to maintaining the flight state of hovering in the present study can provide guidance for the structural design and kinematic control of micro aerial vehicles.

## 1. Introduction

Hovering, defined as flight with zero net velocity relative to the air, is an important flight state for flying animals and artificial aircraft and has fascinated many researchers [1,2,3,4,5,6,7,8]. According to helicopter theory [9], it has been proposed that for revolving wings, the relationship between the power requirement of flight and the flight speed follows a U-shaped function, so that very slow-speed flights (e.g., hovering) require more power than moderate-speed flights. However, based on the measurements of oxygen consumption of bumblebees and hummingbirds in forward flight [10,11], it was concluded that the relationship between mechanical power and flight velocity during flapping flights followed a J-shaped function. The measurements showed that there was little dependence of metabolic power on speed from hovering to moderate speed flight. Simulation data [12] supported this conclusion by investigating the power requirements of fruit flies in forward flight. Hovering with flapping wings has better aerodynamic performance than hovering with revolving wings [13].

According to previous observations [14,15], most kinds of insects (e.g., fruit flies, honeybees, and beetles) hover on a horizontal plane, which was defined as normal hovering. The other kind of hovering, defined as inclined hovering, was mainly observed in true hoverflies and dragonflies [16,17,18,19,20,21]. In these two kinds of typical hovering, the stroke plane angle is 0° for normal hovering and 60° for inclined hovering [16,18,22].

The aerodynamic characteristics of inclined hovering were first studied based on the dragonfly’s flight [23,24,25,26]. By comparing the difference in wing kinematics and instantaneous forces between normal hovering and inclined hovering, the results showed that asymmetric wing kinematics was a significant characteristic of inclined hovering. The insects with normal hovering applied symmetry wing kinematics, in which the wings produced horizontal aerodynamic forces of equal magnitude and opposite directions during the downstroke and upstroke, resulting in the horizontal force balance. While hovering on an inclined stroke plane, as the thrust and lift must be vectorially combined into a vertical force to support the flyer’s weight, the wings need to generate non-zero drag and lift relative to the stroke plane. Therefore, the wing kinematics of inclined hovering needs to be non-symmetric between the downstroke and upstroke. The influence of asymmetric wing kinematics on the aerodynamic performance of inclined hovering is worth investigating.

The study of Wang [26] showed that during inclined hovering, the translational motion of the wing generated a pair of counterrotating vortices at the leading edge and the trailing edge and the dipole vortex jet composed of two counter-rotating vortices during the upstroke played an important role in the vertical force generation. Wang [27] studied the inclined hovering by changing the stroke plane angle in a two-dimensional numerical simulation. The results showed that the inclined hovering allowed the insect to convert some of its translational drag into vertical lift to support its weight during the downstroke. In the case of a dragonfly hovering, the drag supported approximately 76% of its weight. By comparing the relationship between the force coefficient and power with the stroke plane angle, it was found that a stroke plane angle of 60° was a cut-off for vertical force generation, which was consistent with the stroke plane angle of dragonflies. Kim and Choi [28] and Sudhakar and Vengadesan [29] conducted two-dimensional numerical simulations to investigate the effects of rotation timing and rotation speed during inclined hovering. The results showed that as in the study of normal hovering [12,30,31], advanced rotation and fast rotation could increase the lift in inclined hovering. Jardin, et al. [32] proposed a parametrical study of inclined hovering using the model experiment method. Based on the TR-PIV, they obtained the vorticity fields and evaluated the corresponding unsteady forces. The results showed that the inclined hovering had an efficiency advantage over normal hovering flapping. Park and Choi [33] conducted an experiment with a pair of dynamically scaled model wings to investigate the effect of the asymmetry of the angle of attack on the aerodynamic force of inclined hovering. The study suggested that manipulating the angle of attack was the most effective method to control the aerodynamic forces and the power consumption of dragonfly-like inclined flapping. The research of Zhu and Sun [34] on inclined hovering showed that the wing moved rapidly downwards and forward at a large angle of attack during the downstroke, and strong counter-rotating vortices generated by the leading and the trailing edges produced a large rate of change in the first moment of vorticity, which accounted for the large aerodynamic force. Deepthi and Vengadesan [35] investigated the ground effect of inclined hovering. The results showed that the direct jet impingement and ram effect caused by vortex shedding and the dipole jet contributed to the high lift near the ground. Other simulations of the inclined hovering [36,37,38] reached similar conclusions to those of the references above.

In summary, inclined hovering achieves a better aerodynamic performance compared to normal hovering by making use of drag during the downstroke and has a better ground effect due to the vortex shedding and dipole jet. However, most existing Micro Aerial Vehicles (MAVs) conduct normal hovering [39,40,41]. Considering the better aerodynamic performance, it is a better strategy for MAVs to apply inclined hovering. Therefore, to further explore the aerodynamic advantages of inclined hovering, the aerodynamic effects of asymmetric rotation on inclined hovering are systematically studied in this paper. As can be seen from the above summary, most studies on inclined hovering adopt numerical methods and lack the corresponding model experiments. Therefore, the development of a model experiment to study inclined hovering is helpful to understand the physical mechanism of inclined hovering in more detail. In Section 2, a model test bench for the flapping of a hovering dragonfly is designed and assembled, through which the aerodynamic mechanisms of key parameters in inclined hovering are analyzed in Section 3.

## 2. Methodology

### 2.1. The Kinematic Characteristics of Inclined Hovering

**Figure 1:**
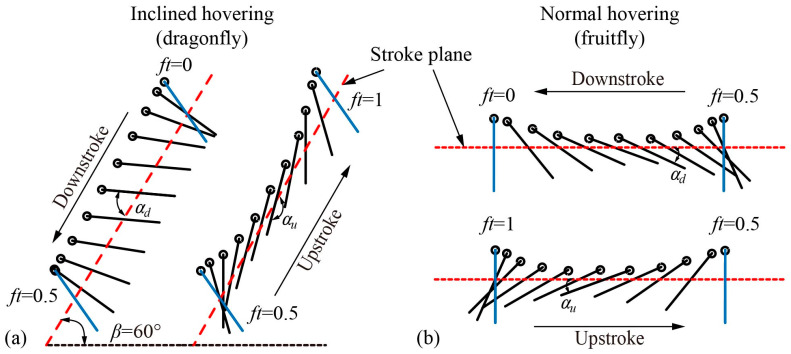
The diagram of wing motion: (**a**) Inclined hovering; (**b**) normal hovering.

Compared to normal hovering, as shown in Figure 1, one distinct kinematic characteristic of inclined hovering is the asymmetry of the rotational angle between the upstroke and the downstroke. In Figure 1, the location of the wing chord at the start and the end of a half stroke is marked in blue with its corresponding dimensionless time within a flapping cycle (*ft,* where *f* is the flapping frequency and *t* is the time) also marked. *ft* = 0 and 0.5 correspond to the start of the downstroke and the upstroke, respectively. The definition of wing kinematics adopted in this paper can be referred to in previous studies [42,43], so it is briefly described here. The stroke plane is defined by the trajectory of the wing tip and the wing root. The stroke plane angle *β* is the angle between the stroke plane (marked as a red dotted line in Figure 1) and the horizontal plane. The translational angle *θ* is defined as the angle between the line from the wing root to the wing tip and the horizontal plane, and the rotational angle *α* is defined as the angle between the wing surface and the stroke plane. *α_d_* is the rotational angle of the mid-downstroke and *α_u_* is the rotational angle of the mid-upstroke. Normal hovering applies a stroke plane parallel to the horizontal plane, and the kinematics of *α* in the upstroke and downstroke are the same (*α_d_* = *α_u_*). While a dragonfly’s inclined hovering applies an inclined stroke plane of 60° from the horizontal plane, the kinematics of *α* in the upstroke and downstroke are asymmetrical (*α_d_* < *α_u_*). Therefore, *α_d_* and *α_u_* are the characteristic parameters of inclined hovering aerodynamics.

According to the observation results of hovering dragonflies [20,44,45], the kinematics of the forewing can be simplified as

(1) $$\theta (t)=\frac{\theta_{m}}{\sin^{-1}(C_{\theta })}\sin^{-1}[C_{\theta }\cos(2\pi ft)]+\theta_{0}$$

where *θ_m_* is the translational amplitude, *C_θ_* is the translation profile parameter, and *θ*_0_ is the average translational angle;

(2) $$\alpha (t)=\frac{\alpha_{m}}{\tanh(C_{\alpha })}\tanh[C_{\alpha }\sin(2\pi ft)]+\frac{\alpha_{d}+\alpha_{u}}{2}$$

where *α_m_* is the rotational amplitude and *C_α_* is the rotation profile parameter. The values of kinematic parameters are summarized in Table 1.

### 2.2. Experiment Setup

**Figure 2:**
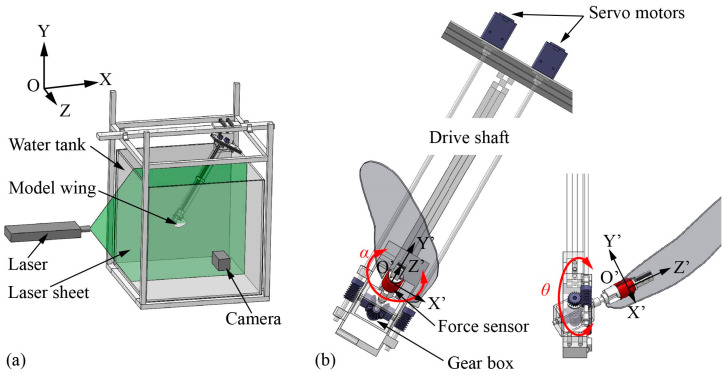
(**a**) Experimental setup; (**b**) side view and front view of the robotic apparatus.

The experiment is conducted in a glass-walled tank with a size of 1000 mm square, as shown in Figure 2a. During the experiment, the tank is filled with water at a depth of 900 mm, and the wing root coincides with the center of the fluid. According to the study by Dickinson, Lehmann, and Sane [30], the changes in the mean lift coefficient with distance from the solid–liquid (side and bottom) and air–liquid (top) interfaces were closely approximated by exponential functions, based on which the wall effect of the experimental setup in this paper produces less than 0.5% of the force in each direction, suggesting that the experimental conditions are well approximated by the infinite volume.

We design and build a dynamically scaled robotic system that can reproduce the dragonfly’s inclined hovering. To achieve precise control of the wing motion, two servo motors (MX-28T, resolution 1/4096°) equipped with high-resolution encoders are selected to drive the machinery, and in-house code written with LabVIEW is used to control the position of the motors with a high temporal resolution (5 ms). As shown in Figure 2a, an inertial coordinate system OXYZ is introduced, where the OXZ plane is the horizontal plane, the positive X-direction points to the posterior direction of the hypothetical, non-existent dragonfly body, the positive Y-direction points vertically upward, and the *Z*-axis is determined by the right-hand law. In addition, a non-inertial coordinate system O′X′Y′Z′ that is fixed on the wing is also introduced, where Point O′ is located at the root of the wing, the X′-axis is parallel to the wing chord direction and its positive direction points to the trailing edge, the Y′-axis is perpendicular to the wing surface and its positive direction points upward, and the Z′-direction is determined by the right-hand law.

**Figure 3:**
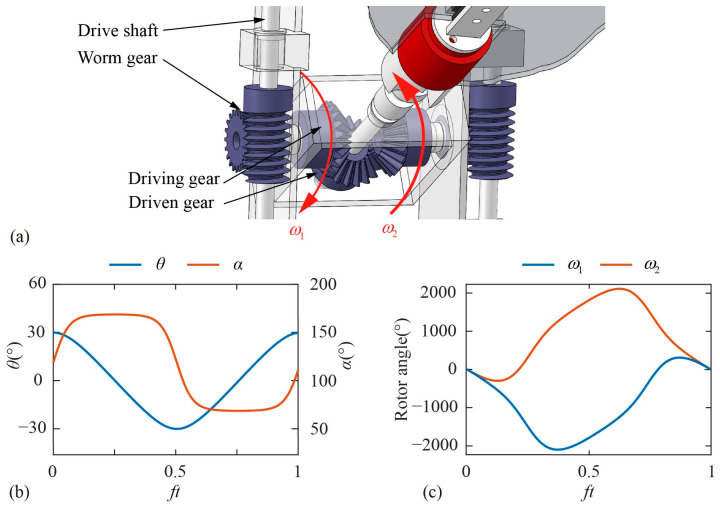
(**a**) The transmission law of gearbox; (**b**) the kinematics of the wing; (**c**) the control of the servo motors.

A special parallel differential gearbox is designed to convert the motion of the servo motors into the translation and rotation of the model wing. As shown in Figure 2b and Figure 3a, the differential gearbox consists of two driving gears, one driven gear and the corresponding supporting structure. The motion of the two servo motors is transmitted to the two driving wheels through the drive shaft and the worm gear, respectively, where the transmission ratio of the worm gear *λ* is 20:1. To ensure transmission accuracy, the gears and support structure of the gearbox are 3D printed with a resolution of 0.05 mm. Different from the Series drive system used in the previous model experiment in which each motor controls one of the kinematic DOFs independently [46,47,48], the two kinematic DOFs of the model wing in the parallel differential transmission used in the present experiment are controlled jointly by two motors, which is beneficial to increase the accuracy and range of motion. The kinematic relationship between the motors and the model wing is as follows:

(3) $$\left\{ \begin{array}{l} \theta (t)=\frac{\omega_{1}(t)-\omega_{2}(t)}{2*\lambda }\\ \alpha (t)=\frac{\omega_{1}(t)+\omega_{2}(t)}{2*\lambda } \end{array}\right.$$

where *ω*_1_ and *ω*_2_ are the speeds of motor 1 and motor 2 as shown in Figure 3a. Figure 3b shows the kinematics of the forewing in dragonfly hovering and Figure 3c shows the corresponding kinematics of the motors. The angle sensor (HWT6073-485, resolution 0.001°) is fixed on the model wing to measure the instantaneous translational angle and rotational angle. The relative standard deviation of the translational angle and the rotational angle are 0.75% and 0.67%, respectively. Related calculations are attached in Supplementary Material.

To ensure that the flow generated by the flapping model wing is aerodynamically similar to that of a dragonfly’s flapping flight, its geometric size and flapping frequency are set according to a certain Reynolds number (*Re* = *V*_tip_*c*/*ν*, where *V*_tip_ is the average translational speed of the wing tip, *c* is the average chord length, and *ν* is the kinematic viscosity coefficient of air). The geometric data of the model wing profile are extracted from the forewing of the dragonfly (*Pantala flavescens*, *Libellulidae*) captured at Beihang University. The model wing is five times the size of the real wing with a span of 174 mm and an average chord length *c* of 35 mm. The model wing is made of optical glass and can be regarded as a rigid wing. The flapping frequency is 0.1 Hz, and *Re* is calculated as 1642. This value for *Re* is consistent with the value observed for dragonflies hovering (1000–2000) [20]. 

### 2.3. Force Measurement

The instantaneous force of the model wing is measured by a six-axis F/T sensor (Nano17-IP68, ATI industrial Automation. Inc., Apex, NC, USA) mounted at the wing root. The sensor can measure the forces in the X′, Y′, and Z′-directions based on its own Lagrange coordinate system as shown in Figure 2b. The range of force measurement in the X′ and Y′-direction is 12 N, and in the Z′-direction it is 17 N, with a resolution of 1/320 N in each direction. The force data are collected at a frequency of 40 kHz with an average level of 200, resulting in an effective sampling rate of 200 Hz, which is 2000 times that of the flapping wing frequency. A sixth-order low-pass Butterworth filter is used to filter the raw data. The cut-off frequency is 2 Hz, which is 20 times that of the flapping wing frequency. The forces measured by the sensor are the resultant force of the aerodynamic force, gravity, buoyancy, and inertia. To obtain the aerodynamic force of the wing, the buoyancy, gravity, and inertia forces need to be removed. During the experiment, the directions and magnitudes of buoyancy and gravity remain constant. Therefore, the buoyancy and gravity can be measured before the experiment and subtracted from the raw data. Inertial force is related to the acceleration and mass of the model wing. Based on the theoretical model [49], the inertial force (~10^−4^ N) of the model wing can be ignored relative to the aerodynamic force (~10^−1^ N) in this experiment.

Lift *L* and thrust *T* are defined as the aerodynamic force perpendicular to and parallel to the horizontal plane, respectively. *F_X′_*, *F_Y′_*, and *F_Z′_* are defined as the aerodynamic forces along the X′, Y′, and Z′-axes of the Lagrange coordinate system, respectively, namely the force data measured and output by the force sensor. *F_X_*, *F_Y_*, and *F_Z_* are the aerodynamic forces along the X-, Y-, and Z-axes of the inertial coordinate system, where *F_X_* is equal to *L*, *F_Y_* is equal to *T*, and *F_Z_* is the lateral force. The coordinate system transformation matrix is applied to the sensor measurements [*F_X′_*, *F_Y′_*, *F_Z′_*] in the Lagrange coordinate system to obtain the wing aerodynamic forces [*F_X_*, *F_Y_*, *F_Z_*] in the inertial coordinate system.

The lift coefficient *C_L_* and the thrust coefficient *C_T_* are calculated as:

(4) $$C_{L}=\frac{L}{0.5\rho SV_{tip}^{2}},\;C_{T}=\frac{T}{0.5\rho SV_{tip}^{2}}$$

where *ρ* is the density of the fluid and *S* is the area of the wing.

The uncertainty of the lift coefficient in the experiment system bounded at 95% (2σ) is calculated as 1.43%. Related calculations are attached in Supplementary Material.

### 2.4. PIV Measurement

**Figure 4:**
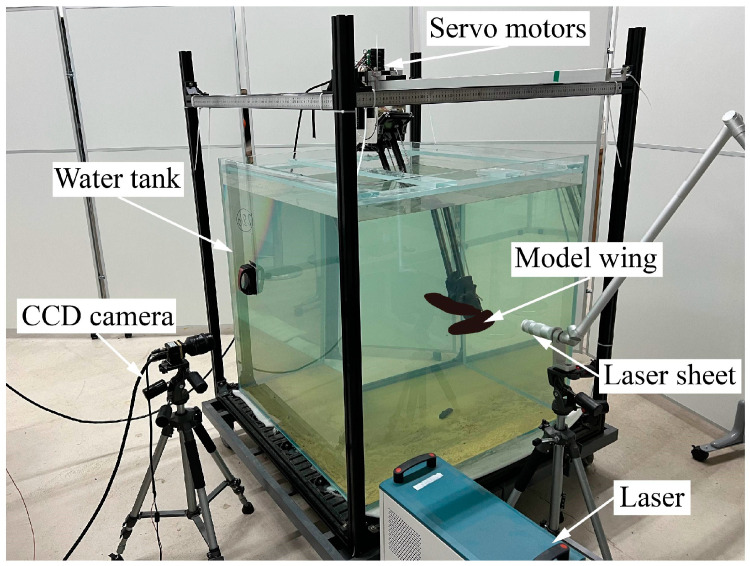
Photograph of the experimental setup.

To measure the instantaneous flow fields of the flapping wing, a customized Particle Image Velocimetry (PIV) [50,51] system (DM3-5M200, MicroVec. Inc., Beijing, China, https://www.piv.com.cn/) is adopted in this experimental system. Figure 4 shows the layout of the components and experimental devices of the PIV system. A high-speed CCD camera (SM-CCDB5M16) is equipped with a professional optical lens (NIKON 24 mm/F2.8), providing a measurement field of view with a pixel resolution of 2456 (H) × 2056 (V) and a size of 350 mm × 300 mm. The high-energy dual-pulse laser (SM-LASER-BM200-15) provides a laser with an energy of 200 mJ and a wavelength of 532 nm. The optical element composed of a convex mirror and a concave mirror can convert the laser beam into a laser sheet with a width of less than 1 mm. The PIV synchronizer (SM MicroPulse725) uses a TTL signal to trigger the laser and CCD camera to work in synchronization with a time resolution of 0.25 ns. Hollow glass particles with a diameter of 10 μm are seeded into water as tracer particles. The sinking velocity of a sphere under laminar flow can be expressed by the Stokes Formula, $u_{\infty }=gd_{p}^{2}(\rho_{p}-\rho_{f})/(18\mu )$, where *g* is the acceleration due to gravity, *d_p_* is the particle diameter, *ρ_p_* is the particle density, *ρ_f_* is the fluid density, and *μ* is the fluid viscosity. The sinking velocity of tracer particles in this experiment is ~10^−5^ m/s, which is close to zero and much less than the average velocities of the flow (~10^−1^ m/s). Therefore, the tracer particles are acceptable for PIV measurement.

The CCD camera is positioned perpendicular to the laser sheet. The dual-pulse laser can produce a pair of laser sheets at a specific time interval. The CCD camera can capture a pair of images corresponding to the flow fields illuminated by two laser sheets. Using cross-correlation calculation of this pair of images, the displacement information of the same particle in the time interval can be obtained. By adjusting the position of the laser sheet and the trigger time of the PIV measurement, the flow fields at different spanwise sections of the model wing during flapping can be obtained. The software Micro Vec V3 (Micro Vec. Inc., Singapore) is used to post-process the flow field. The software applies sub-pixel Gaussian curve fitting to increase the calculation accuracy of the result to ±0.1-pixel accuracy and corrects the error vector through the median filter algorithm to obtain high-precision flow field information. Compared with the velocity distribution of the artificial rotation velocity field in the numerical simulation, the relative error of the software system is less than 1%.

### 2.5. Temporal Procedure for the Experiment

**Figure 5:**
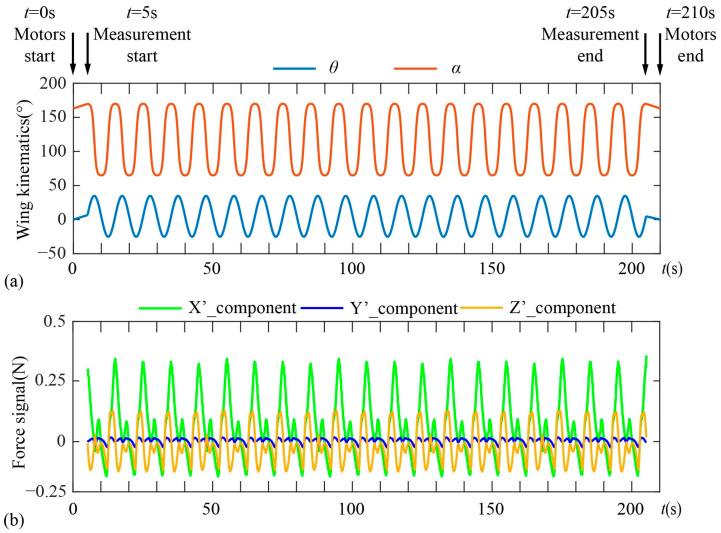
(**a**) Wing kinematics and (**b**) force signal during the experiment.

Figure 5 shows the temporal procedure of the experiment. To ensure that the wing is in the same initial position at the beginning of each experiment, the wing is adjusted to the horizontal plane by controlling the motors before the experiment. At the beginning of the experiment (*t* = 0 s), the system program sends a trigger signal to drive the servo motors to move the model wing from the horizontal plane to the initial flapping position. After half the flapping cycle (*t* = 5 s), the system program controls the servo motors to drive the model wing to flap periodically, and at the same time sends a trigger signal to control the sensor to measure the force. When *t* = 205 s, the model wing has completed 20 cycles of flapping and starts to move from the end position of flapping to the horizontal plane, and the force sensor stops recording data at the same time. Finally, when *t* = 210 s, the model wing moves to the horizontal plane. Each experiment lasts 21 flapping cycles, which equals 210 s in time.

The time interval between the two adjacent groups of experiments is more than 2 min to ensure that the wake generated by the previous experiment is completely dissipated before the start of this experiment. Since the flow field is unstable at the beginning and end of flapping, the force data of 15 consecutive cycles from the 4th to the 18th are averaged to obtain the time course of aerodynamic force within a cycle.

### 2.6. Validation

**Figure 6:**
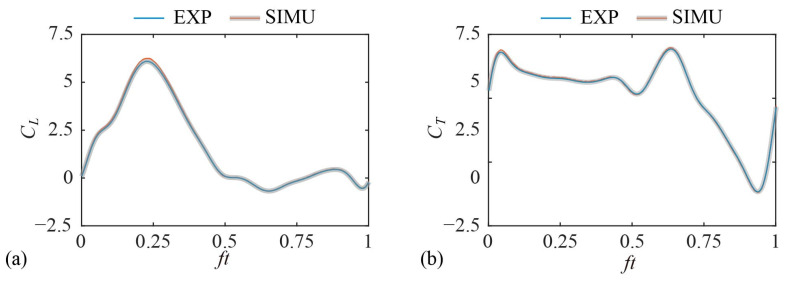
Validation by comparison with simulation results: (**a**) lift coefficient; (**b**) thrust coefficient.

In order to evaluate the accuracy of the experimental method, the measured aerodynamic force production by the forewing during hovering is compared to that obtained by a numerical Lattice–Boltzmann Method [42,43]. The uncertainty of the force coefficients measured by the experimental method (see Supplementary Material) is denoted as the gray ribbon in Figure 6. As shown in Figure 6, the force coefficient obtained by the two methods are in good agreement. The average lift coefficients of the model experiment and the numerical simulation are 1.59 and 1.64, respectively. The average thrust coefficients of the model experiment and numerical simulation are 0.11 and 0.12, respectively. The root-mean-square error of the lift coefficient and the drag coefficient are within 4% of the simulation results.

## 3. Results and Discussion

According to the analysis in Section 2.1, *α_d_* and *α_u_* are the characteristic parameters of inclined hovering. Therefore, this paper applies the model experiment method to investigate the aerodynamic mechanisms of the above characteristic parameters on inclined hovering. The kinematic parameters of the benchmark case are the same as those of the forewing in dragonfly hovering as defined in Section 2.1. Based on the principle of a single variable, there are two groups of experiments: Group 1 investigates the effect of *α_d_* on aerodynamics and Group 2 investigates the effect of *α_u_* on aerodynamics. The parameters of the experiment are shown in Table 2.

### 3.1. Characterization of Inclined Hovering with Different α_d_

#### 3.1.1. Time Courses of Aerodynamic Force

**Figure 7:**
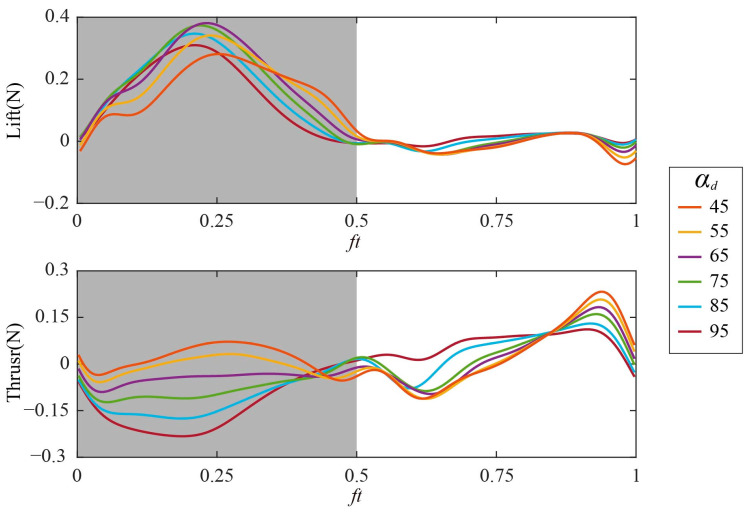
Time courses of aerodynamic forces with different *α_d_*: (**top**) Lift; (**bottom**) thrust.

According to the description of the coordinate system transformation and experimental process in Section 2.3 and Section 2.5, the time courses of the lift and thrust of Group 1 are shown in Figure 7, where the gray box represents the downstroke. *α_d_* mainly affects the aerodynamic force in the downstroke (*ft* = 0–0.5). During the downstroke, the lift first increases and then decreases, and the maximum instantaneous lift *L*_max_ is obtained near the mid-downstroke (*ft* = 0.25). During the upstroke (*ft* = 0.5–1), almost no lift is generated. When *α_d_* increases from 45° to 95°, *L*_max_ increases first and then decreases, reaching its maximum value when *α_d_* = 65°. The thrust in the downstroke gradually decreases as *α_d_* increases from 45° to 95°.

**Figure 8:**
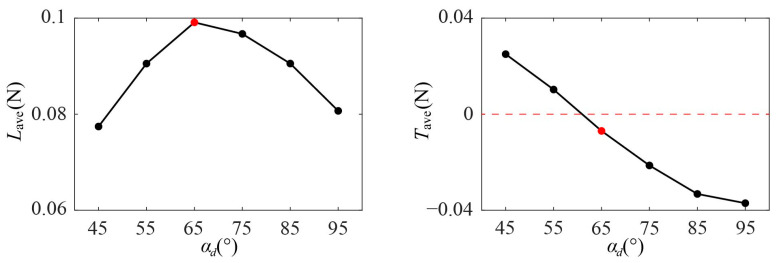
The cycle-averaged aerodynamic forces of Group 1.

According to the summary of the cycle-averaged aerodynamic forces of Group 1 in Figure 8, when *α_d_* increases from 45° to 95°, the average lift *L*_ave_ first increases and then decreases, reaching the maximum value at *α_d_* = 65°. The average thrust *T*_ave_ decreases gradually as *α_d_* increases from 45° to 95° and changes from positive to negative near when *α_d_* = 65°. During hovering, the dragonfly’s center of mass is at rest relative to the air, so the resultant force is zero, which means the wing needs to provide positive lift to balance the body weight and an average thrust close to zero. According to the above force analysis, when *α_d_* = 65° (as marked by the red circle in Figure 8), *L*_ave_ is the maximum (0.0991 N) and *T*_ave_ is the closest to zero (0.0070 N), which is conducive to the maintenance of hover.

#### 3.1.2. Vortex Structure at Mid-Downstroke

The effect of *α_d_* on the aerodynamic force is most significant in the mid-downstroke (*ft* = 0.25). Therefore, the flow fields of the wing’s characteristic section *r*_2_ at the characteristic time *ft* = 0.25 are measured by PIV to investigate the aerodynamic mechanism of *α_d_*. *r*_2_ is the radius of the second moment of the model wing and is defined as

(5) $$r_{2}=\frac{1}{S}\int_{0}^{R}r^{2}dS$$

where *r* is radial distance, *S* is the area of the forewing, and *R* is the wing length. *r*_2_ = 0.60*R* in this paper.

**Figure 9:**
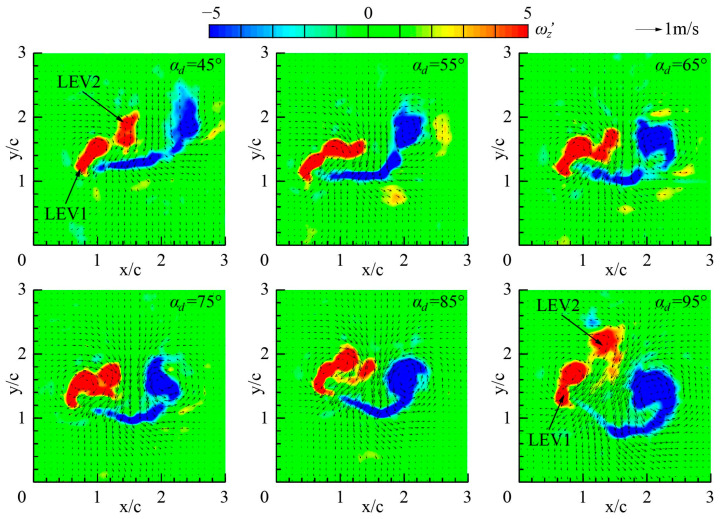
The flow fields of *r*_2_ section in Group 1 at mid-downstroke (*ft* = 0.25).

Figure 9 shows the dimensionless vorticity and the velocity vectors of Group 1. The Z-component of the dimensionless vorticity *ω_z_*′ is defined as *ω_z_*′ = *ω_z_c*/*V*_tip_, where *ω_z_* is the Z-component of the vorticity. When *α_d_* = 45°, the structure of the leading edge vortex (LEV) breaks down in the mid-downstroke, resulting in a new LEV1 near the leading edge and a LEV2 that separates from the wing. When *α_d_* = 95°, the LEV breakage phenomenon is similar to that when *α_d_* = 45°. Therefore, the structure of LEV is very sensitive to *α_d_* in the mid-downstroke. When the value of *α_d_* is too large (*α_d_* = 85°–95°) or too small (*α_d_* = 45°), the angle between the wing surface and the horizontal plane is large in the mid-downstroke, which is not conducive to the stability of the LEV structure and will lead to the breakage of LEV. When *α_d_* is in the appropriate range (*α_d_* = 55°–75°), the wing surface is approximately parallel to the horizontal plane in the mid-downstroke, keeping the structural integrity of the vortex at mid-downstroke.

**Figure 10:**
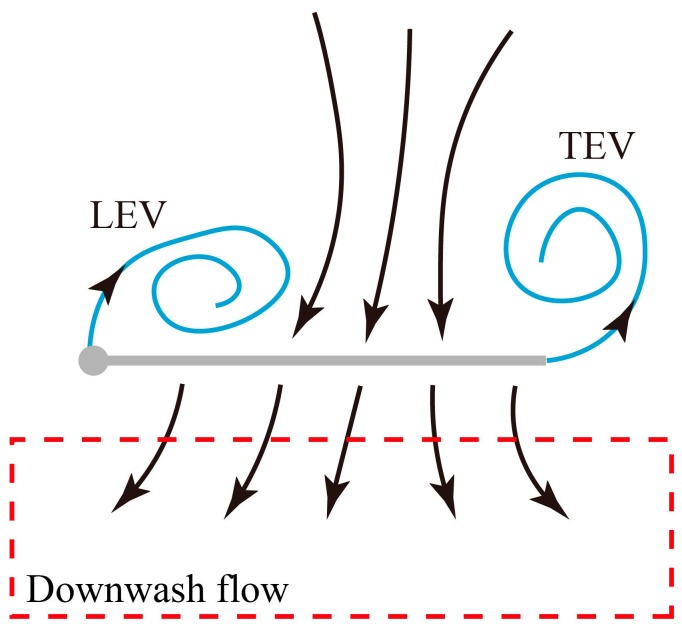
The vortex structure diagram at mid-downstroke.

As can be seen from the velocity vector fields in Figure 9 and the vortex structure diagram in Figure 10, the LEV and the trailing edge vortex (TEV) generate strong downwash airflow near the wing in the mid-downstroke, which is the main cause of the lift. The structural integrity and magnitude of LEV and TEV determine the ability to generate lift during the downstroke of inclined hovering. At mid-downstroke, when the angle between the wing surface and the horizontal plane is less than 15° (*α_d_* = 55°–75°), the LEV and TEV can maintain the structural integrity and large magnitude, which is conducive to the generation of high lift force. The above flow phenomenon explains the large *L*_ave_ obtained when *α_d_* = 55°–85° in Figure 8 (left).

### 3.2. Characterization of Inclined Hovering with Different α_u_

#### 3.2.1. Time Courses of Aerodynamic Force

**Figure 11:**
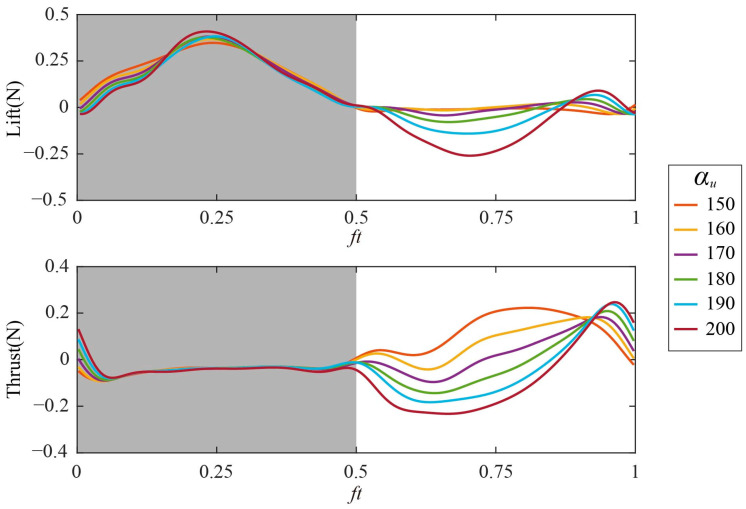
Time courses of aerodynamic forces with different *α_u_*: (**top**) Lift; (**bottom**) thrust.

**Figure 12:**
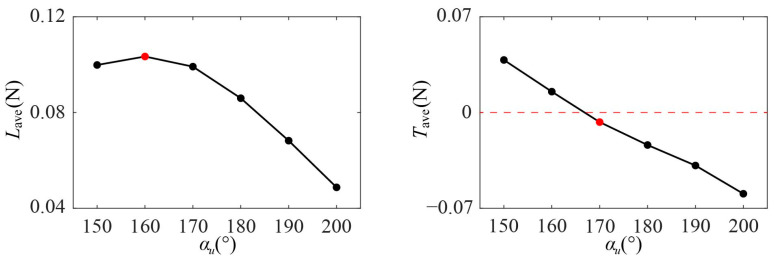
The cycle-averaged aerodynamic forces of Group 2.

The time courses of lift and thrust of Group 2 are shown in Figure 11. α_u_ mainly affects the aerodynamic force in the upstroke (*ft* = 0.5–1). When α_u_ increases from 150° to 200°, both the lift and thrust in the upstroke gradually decrease, while there is little change in the lift and thrust of the downstroke. According to the summary of the cycle-averaged aerodynamic forces of Group 2 in Figure 12, the wing produces a large average lift *L*_ave_ (0.0992 N–0.1034 N) when α_u_ ranges from 150° to 170°, and reaches the maximum *L*_ave_ at α_u_ = 160° (as marked by the red circle in Figure 12). When α_u_ increases from 150° to 200°, the average thrust *T*_ave_ decreases gradually, and changes from positive to negative around α_u_ = 170°. Therefore, according to the force analysis of hovering flight in Section 3.1.1, α_u_ = 160°–170° is conducive to maintaining the hovering state.

#### 3.2.2. Vortex Structure at Mid-Upstroke

To reveal the aerodynamic mechanism of the change in thrust direction as *α_u_* increases from 150° to 200°, the flow fields in the mid-upstroke of *α_u_* = 150°, *α_u_* = 180°, and *α_u_* = 200° are analyzed. 

**Figure 13:**
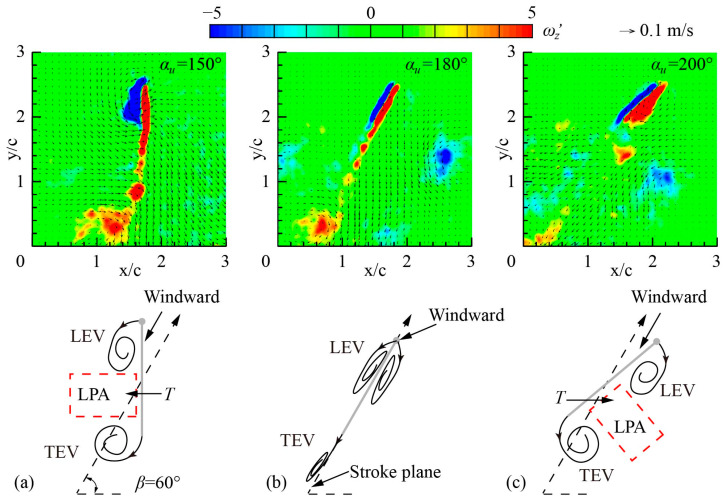
The flow fields of *r*_2_ section in Group 2 at mid-upstroke (*ft* = 0.75).

The maximum forward thrust is obtained when *α_u_* = 150°. Figure 13a shows the flow fields of *r*_2_ section in the mid-upstroke when *α_u_* = 150°. The windward side of the wing in the mid-upstroke when *α_u_* = 150° is defined as the upper surface, and the corresponding wing surface is defined as the lower surface. The LEV and TEV are generated from the leading and trailing edges of the wing, respectively, and attached to the lower surface. This pair of vortices induces the airflow around the wing to accelerate and creates a low-pressure area (LPA) near the lower surface, generating a forward thrust toward the LPA.

The thrust is closest to zero near when *α_u_* = 180°. Figure 13b shows the flow fields of *r*_2_ section in the mid-upstroke when *α_u_* = 180°. The wing surface is parallel to the stroke plane, and the leading edge of the wing is the windward side. The airflow is evenly distributed by the leading edge to the upper and lower surfaces so that two LEVs of the same magnitude and opposite directions are attached to the upper and lower surfaces of the wing, respectively. As a result, almost no thrust is generated during the upstroke.

The maximum negative thrust is obtained when *α_u_* = 200°. Figure 13c shows the flow fields of *r*_2_ section in the mid-upstroke when *α_u_* = 200°. Compared with the flow field when *α_u_* = 150°, the windward surface changes into the lower surface, and the attachment surface of the LEV and TEV changes to the upper surface. The LPA generated by the LEV and TEV is also transferred to the upper surface, causing the wing to produce negative thrust.

The aerodynamic effect of *α_u_* on thrust can be summarized as the windward conversion mechanism: when *α_u_* < 180°, the upper surface of the wing is the windward side during the upstroke, and the LEV and TEV are attached to the lower surface, producing a large forward thrust. When *α_u_* = 180°, the wing surface is parallel to the stroke plane, and the leading edge of the wing is the windward side. Two LEVs of the same magnitude and opposite directions are attached to the upper and lower surfaces of the wing, respectively, generating a thrust close to 0 N. When *α_u_* > 180°, the lower surface of the wing is the windward surface during the upstroke, and the LEV and TEV are attached to the upper surface, resulting in a large negative thrust. Therefore, the dragonfly can actively adjust *α_u_* to cope with different flight states and adjust the direction and magnitude of thrust by changing the windward side.

### 3.3. The Three-Dimensional Vortex Structure of Inclined Hovering

#### 3.3.1. The Three-Dimensional Vortex Structure of Group 1 at Mid-Downstroke

**Figure 14:**
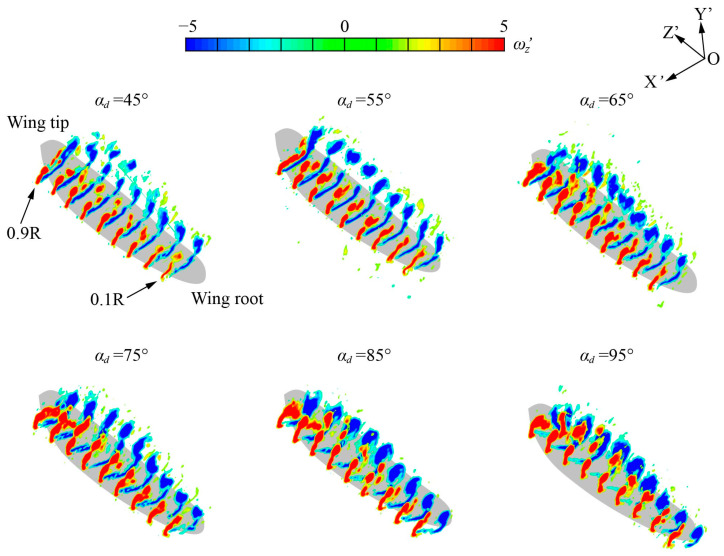
Three-dimensional vortex structure of Group 1 at mid-downstroke (*ft* = 0.25).

The PIV equipment used in this experiment can measure a two-dimensional flow field. Although the two-dimensional flow field of the *r*_2_ section can reflect the flow characteristics of the flapping wing [24,52,53], the flapping has three-dimensional characteristics due to the different velocities in different spanwise sections. Therefore, the two-dimensional vortex structures at the wingspan position of 0.1*R* to 0.9*R* in each experiment of Group 1 are measured to obtain the three-dimensional flow field slices in the mid-downstroke as shown in Figure 14. 

In the mid-downstroke, the LEV and TEV present significant spanwise distribution characteristics: from 0.1*R* to 0.4*R*, the vortex structure is in the development stage of increasing magnitude; from 0.5*R* to 0.7*R*, the vortex structure is in a stable stage with a large magnitude; from 0.8*R* to 0.9*R*, the vortex structure is in an unstable stage of structural decomposition. In this paper, the *r*_2_ = 0.6*R* section is selected for two-dimensional flow field analysis, which is in the stable stage and can represent the aerodynamic characteristics of the flapping flight.

#### 3.3.2. The Three-Dimensional Vortex Structure of Group 2 at Mid-Upstroke

**Figure 15:**
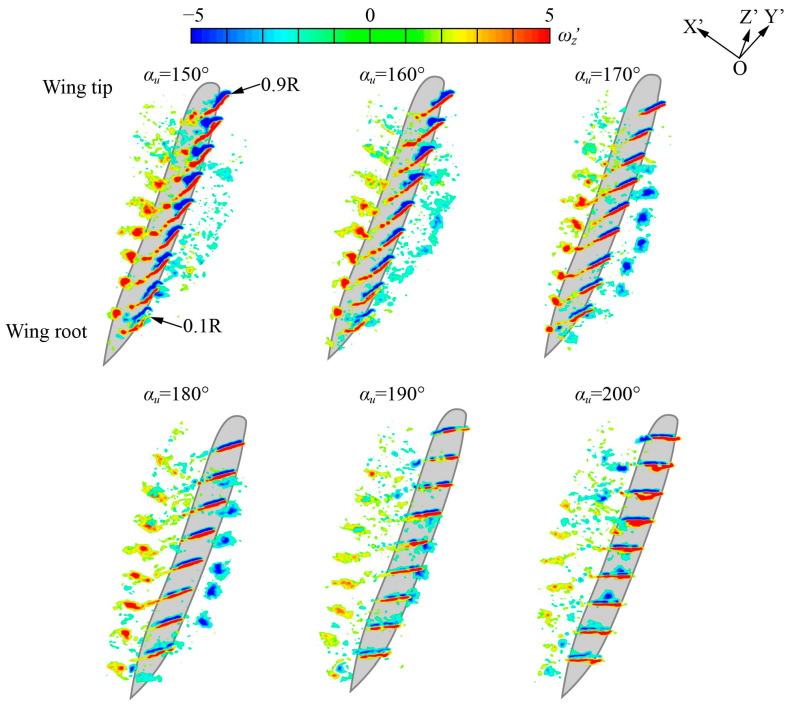
Three-dimensional vortex structure of Group 2 at mid-upstroke (*ft* = 0.75).

Figure 15 shows the three-dimensional flow field of Group 2 at mid-upstroke (*ft* = 0.75). When *α_u_* = 150°–160°, the magnitude of the LEV gradually increases during the development from 0.1*R* to 0.9*R*, maintaining a relatively stable structure and remaining attached to the lower surface of the wing. When *α_u_* = 170°–190°, the wing surface is approximately parallel to the stroke plane, resulting in two LEVs of the same magnitude and opposite directions, one of which is on the upper wing surface and the other on the lower wing surface. During the development from 0.1*R* to 0.9*R*, the two LEVs are less affected by the spanwise position and remain stably attached to the wing surface. When *α_u_* = 200°, the LEV maintains a relatively stable structure and remains attached to the upper surface of the wing. The magnitude of LEV increases gradually during the development from the wing root to the wing tip.

In conclusion, the LEV is more affected by the spanwise position in the mid-downstroke: in Group 1, the LEV structure presents significant spanwise distribution characteristics and breaks down at the wing tip. Meanwhile, the LEV is less affected by the spanwise position in the mid-upstroke: in Group 2, the magnitude of the LEV increases slightly as it develops from 0.1*R* to 0.9*R*, during which the LEV maintains structural stability and stays attached to the wing surface.

## 4. Conclusions

To experimentally investigate the aerodynamic performance of inclined hovering, this study presents a model experiment method that can accurately reproduce the flapping motion of an insect wing and measure the unsteady aerodynamic force and flow field produced by the flapping wing. A dynamically scaled robotic system with a special parallel differential gearbox is designed to accurately convert the motion of two servo motors into the translation and rotation of the model wing, with the relative standard deviation of the output translational angle and the rotational angle being 0.75% and 0.67%, respectively. The instantaneous aerodynamic force produced by the flapping model wing is measured by six-axis F/T sensors, with instantaneous flow fields measured by a customized Particle Image Velocimetry system.

Using the aforementioned method, it is revealed that the wing rotational angle, as characterized by *α_d_* and *α_u_*, significantly affects the flapping motion’s aerodynamic force production and the resultant flow field, especially in the middle of the downstroke and the upstroke. For either the downstroke or the upstroke, the time-averaged lift changes almost quadratically with a varying wing rotational angle, whereas the time-averaged thrust changes almost in a linear manner. When *α_d_* = 65° and *α_u_* = 170°, the maximum lift and the thrust that is closest to zero is produced, which is most conducive to maintaining the flight state of hovering.

*α_d_* and *α_u_* affect aerodynamic force production by changing the instantaneous flow structure. During the downstroke, the LEV and TEV can maintain structural integrity and are of a large magnitude when *α_d_* = 55°–75°, creating a strong downwash flow that generates large lift. During the upstroke, the αu affects thrust generation by changing the location of the low-pressure area on the wing surface. The LEV is more affected by the spanwise position at the mid-downstroke: the magnitude of the vortex structure increases along the spanwise direction near the wing root and maintains a large magnitude until the wing tip, where the vortex structure becomes unstable and broken. The LEV is less affected by the spanwise position in the mid-upstroke: the magnitude of the LEV increases slightly as it develops from the wing root to the wing tip, during which the LEV maintains structural stability and is attached to the wing surface.

On the basis of the present results, one can use the experimental method developed in the present study to further aerodynamically investigate the flapping motion of multiple elastic wings, which better conforms to the real flight conditions of dragonflies. Such studies will be crucial for the better development of corresponding biomimetic micro air vehicles.

## Figures and Tables

**Table 1:** The values of kinematic parameters.

*θ_m_*	*C_θ_*	*θ*_0_	*α_m_*	*C_α_*	*α* *_d_*	*α_u_*
30°	0.8	0°	52.5°	2.5	65°	170°

**Table 2:** Kinematic parameters of the experiment.

Group	ID	*α* *_d_*	*α_u_*
Benchmark	0	65°	170°
Group 1	1–6	45°, 55°, 65°, 75°, 85°, 95°	170°
Group 2	7–12	65°	150°, 160°, 170°, 180°, 190°, 200°

## Data Availability

Available upon request to interested researchers.

## Supplementary Materials

The supporting information can be downloaded at: https://www.mdpi.com/article/10.3390/biomimetics9040225/s1.
