# Supplementary material for: Experimental Investigation on Aerodynamic Performance of Inclined Hovering with Asymmetric Wing Rotation
Source: Biomimetics (Basel). 2024 Apr 9;9(4):225. doi: 10.3390/biomimetics9040225 (PMC11047828; doi:10.3390/biomimetics9040225)
Supplement: Supplementary file 1 [file biomimetics-09-00225-s001.zip › biomimetics-2912338-supplementary.pdf]

SI. The calculation of the relative standard deviation of the angle data

To verify the motion accuracy of the mechanical device, the angle sensor (HWT6073-485, with resolution of 0.001°) is fixed on the model wing to measure the translational angle and the rotational angle. The model wing is driven to flapping for 20 consecutive cycles. The range of translational angle is ±30°, following the sine law; and the range of rotational angle is ±60°, following the trapezoidal law. Since the flow field is unstable at the beginning and end of flapping, the angle data of 15 consecutive cycles from the fourth to the 18th are obtained for error analysis. The measurement data of the translational angle and the rotational angle in these 15 cycles are shown in **S 1**.

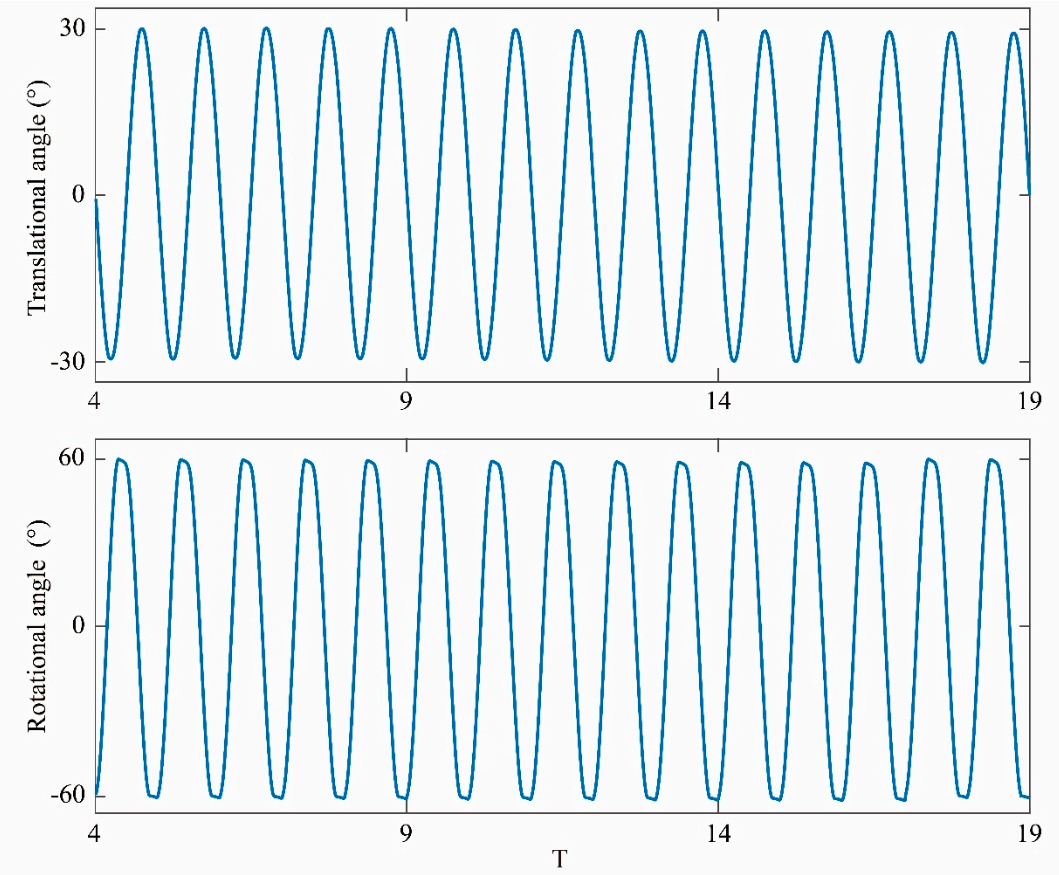

S 1 The measurement data of angle sensor

Each cycle is evenly divided into 100 phase points. The angle sensor measures the translational angle and the rotational angle at each phase point. Calculate the standard deviation of the translational angle and the rotational angle of each corresponding phase point in the 15 cycles, defined as

$$\sigma_j = \sqrt{\frac{\sum_{i=1}^{15}(x_{ij} - \overline{x}_j)^2}{15}}, \quad \text{(S1)}$$

where σ_j represents the standard deviation of the jth phase, x_{ij} represents the measured value of the jth phase in the ith cycle, and $\bar{x}_j$ represents the standard value at the jth phase. Then the standard deviation of the whole cycle σ_{cycle} is defined as

$$\sigma_{cycle} = \sqrt{\frac{\sum_{i=1}^{100}(\sigma_j)^2}{14}}. \quad \text{(S2)}$$

The relative standard deviation of the whole period $\sigma_{relative}$ is defined as the standard deviation σ_{cycle} divided by the maximum absolute value of the measured value $|x_{ij}|_{\max}$:

$$\sigma_{relative} = \frac{\sigma_{cycle}}{|x_{ij}|_{\max}} . \quad \text{(S3)}$$

The $\sigma_{relative}$ of the translational angle and the rotational angle are 0.75% and 0.67% respectively, which verifies the accuracy and periodicity of the mechanical device.

SII. The calculation of the relative standard deviation of the force data

In the benchmark example of the model experiment, the force sensor measures the force of the dragonfly's forewing in hover flight, and obtains the force of the wing in the X ', Y 'and Z' directions during the 20 flapping cycles. Since the flow field is unstable at the beginning and end of flapping, the force data of 15 consecutive cycles from the fourth to the 18th are selected to calculate the force measurement error as shown in **S 2**.

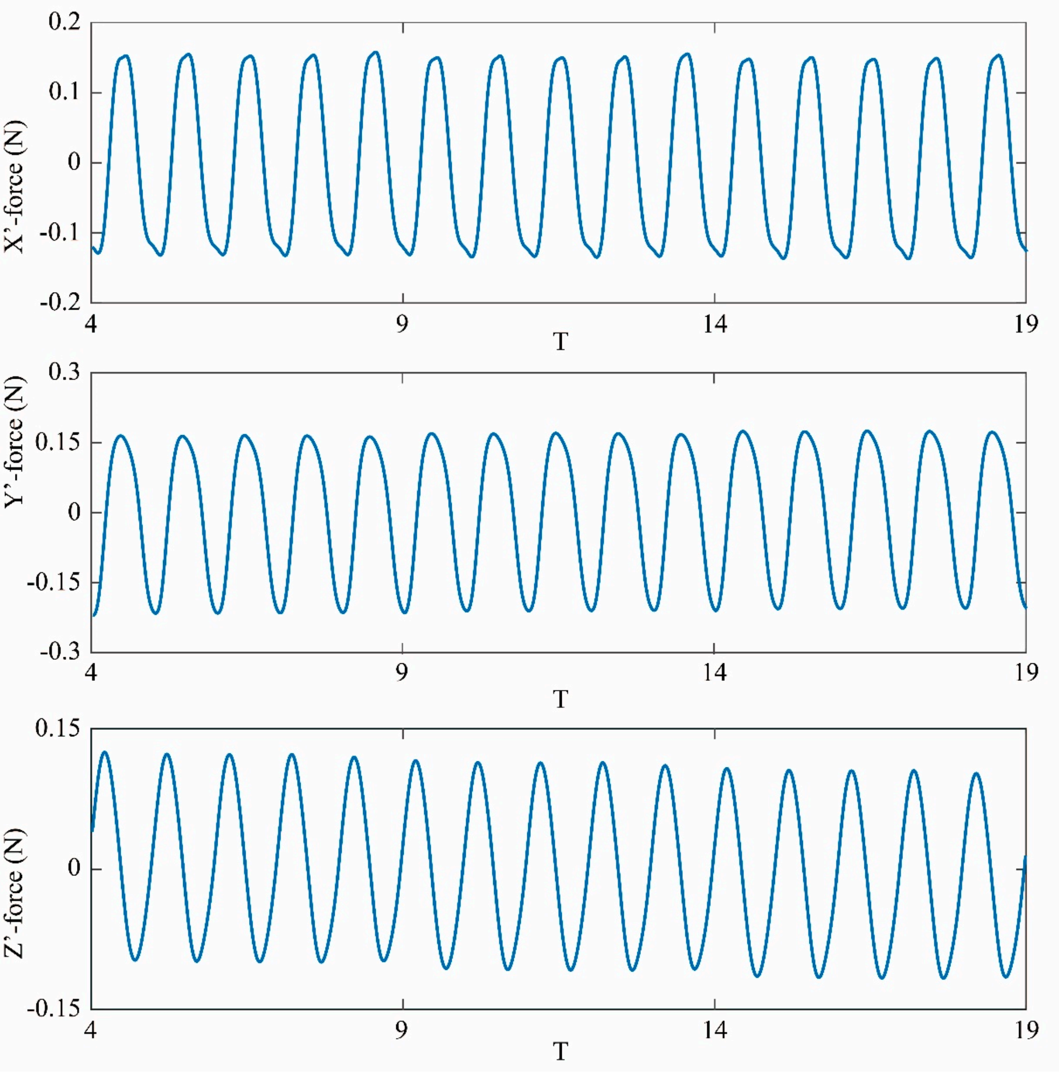

S 2 The measurement data of force sensor

SIII. Uncertainty Analysis

The uncertainty U is generally used to indicate the closeness of the agreement between an experimentally determined value of a quantity and its true value. In this experiment, the uncertainty analysis of lift coefficient is made at the 95% confidence level, which means that the true value of the quantity is expected to be within the $\pm U$ interval about the experimentally determined value 95 times out of 100.

When measuring physical quantities, the experimental system is subject to many error sources. Errors can be composed of two components: If an error contributes to the scatter of the data, it is a precision error P; otherwise, it is a bias error B.

As for precision error, the magnitude of the precision error in measurement of a quantity X_i is defined as P_i, which is given by $P_i=K\sigma_i$. K is the coverage factor and equals 2 for the 95% confidence level, σ_i is the standard deviation of the quantity X_i, which is defined in **Eq.(S1)**.

When the experimental variable obtained in the experiment are represented by multiple measurement quantities, the relationship between the uncertainties of the multiple measurement quantities on the right side of the equation and the uncertainty of the experimental variable on the left side of the equation can be determined by the uncertainty transfer formula. Assume that N is the experimental variable, X_i are the measurement quantities, and $N=f(X_1, X_2, \dots, X_J)$. The precision limit of experimental variable P_N is calculated as follows:

$$P_N=[\sum_{i=1}^{J}(\theta_i P_i)^2]^{\frac{1}{2}}, \tag{S4}$$

where $\theta_i = \partial f/\partial X_i$.

The lift coefficient C_L is defined as

$$C_L=\frac{2L}{\rho U^2 S}=\frac{2L}{\rho(2\Phi fR)^2 S}=\frac{2f(F_{X'},F_{Y'},F_{Z'})}{\rho(2\Phi fR)^2 S}. \tag{S5}$$

Based on the definition of **Eq.(S4)** and **Eq.(S5)**, the precision limit of lift coefficient is composed of the following items: the precision error of force measurement in X ', Y' and Z 'direction; the precision error of the translational angle and the rotational angle; the error of the fluid density ρ due to room temperature change; the error of the flapping frequency f.

Based on the calculation in **Section SI** and **Section SII**, the precision error of force measurement in X 'direction is 0.45%, Y' direction is 0.47%, and Z 'direction is 0.75%. The precision error of the translational angle and the rotational angle are 0.75% and 0.67% respectively. During the experiment, the room temperature range is 24°C±2°C, the water density range is 997.30±0.49kg/m3, and the density error is 0.049%. The angle position of the servo motor is updated every 5 milliseconds, 2000 times per cycle. Therefore, the error of the flapping frequency is 1/2000=0.2%. Based on the above calculations and the definition of precision limit in **Eq.(S4)**, the precision limit of lift coefficient $P(C_L)$ is 1.43%. The calculation process is shown as:

$$\begin{aligned}P(C_L)&=\sqrt{[\frac{\partial F}{\partial F_x}U(F_x)]^2+[\frac{\partial F}{\partial F_y}U(F_y)]^2]^2+[\frac{\partial F}{\partial \alpha}U(\alpha)]^2+[\frac{\partial F}{\partial \theta}U(\theta)]^2+[\frac{\partial F}{\partial \rho}U(\rho)]^2+[\frac{\partial F}{\partial f}U(f)]^2}\\&=\sqrt{(0.45)^2+(0.47)^2+(0.75)^2+(0.75)^2+(0.67)^2+(0.049)^2+(0.2)^2}\\&=1.43\%\end{aligned}. \tag{S6}$$

As for bias error, assume that the measurement variable X_i has multiple bias limits $((B_i)_1, (B_i)_2, \dots, (B_i)_M)$, then the bias limit of the measurement variable B_i is calculated as the root mean square of each bias error:

$$B_i = [\sum_{k=1}^{M} (B_i)_k^2]^{\frac{1}{2}}. \quad \text{(S7)}$$

In this experiment, the bias error consists of the following components: the stroke plane angle, data acquisition system and data processing. The bias error of the stroke plane angle is caused by the calibration error of the scale when installing the mechanism. The precision of the scale is 5' and the bias error of the stroke plane angle is 5'/360°=0.02315%. The bias error of the data acquisition system is caused by the systematic error of the data acquisition (DAQ) card. The resolution of the DAQ card adopted in this experiment is 16 bits, and the bias error is $1/2^{16}$=0.0015%. The bias error of data processing process is caused by the error of data processing software. The software adopted in this experiment is 64-bit MATLAB and LabVIEW. The bias error is $1/2^{64}=10^{-20}$%, which can be negligible. So, the bias limit $B(C_L)$ can be calculated as $\sqrt{0.02315\%^2 + 0.0015\%^2} = 0.023\%$.

Therefore, the uncertainty of lift coefficient can be calculated as

$$U(C_L) = \sqrt{(P(C_L))^2 + (B(C_L))^2} = 1.43\% \quad \text{(S8)}$$
